# Manoeuvring between interplay and context- an ethnographic study of social interaction in encounters between registered nurses, older patients and their relatives

**DOI:** 10.1186/s12912-021-00754-5

**Published:** 2021-11-18

**Authors:** Anette Johnsson, Åse Boman, Petra Wagman, Sandra Pennbrant

**Affiliations:** 1 Department of Health Sciences, University West, Gustava Melins Gata 2, SE-461 86 Trollhättan, Sweden; 2 Department of Rehabilitation, Jönköping University, Jönköping, Sweden

**Keywords:** Abduction, Care encounters, Ethnography, Goffman’s interactional perspective, Interplay, Social interaction, Thematic analysis

## Abstract

**Background:**

Social interactions between registered nurses, older patients and their relatives are essential and play a central role in developing a successful care relationship in healthcare encounters. How nurses interact with patients affects the patient’s well-being. Limited time and demands for efficiency influence the encounter and complaints from patients and relatives often concern social interactions. Therefore, the aim of this study was to explore the social interaction in encounters between registered nurses, older patients and their relatives at a department of medicine for older people.

**Methods:**

The study has an ethnographic approach including participatory observations (*n* = 21) and informal field conversations (*n* = 63), followed by a thematic analysis with an abductive approach reflecting Goffman’s interactional perspective.

**Result:**

The result revealed a pattern where the participants manoeuvred between interplay and context. By manoeuvring, they defined roles but also created a common social situation. Nurses led the conversation; patients followed and described their health problems, while relatives captured the moment to receive and provide information. Finally, nurses summarised the encounter using ritual language, patients expressed gratitude through verbal and non-verbal expressions, while relatives verbally confirmed the agreements.

**Conclusion:**

The social interaction between registered nurses, older patients and relatives was shaped by a pattern where the participants manoeuvred between interplay and context. When all participants assume responsibility for the social interaction, they become active and listen to each other. The approach adopted by nurses is crucial, thus training in communication and social interaction skills are important. When the asymmetry due to imbalance, is reduced, less misunderstanding and a satisfactory care relationship can be achieved.

## Background

Worldwide, social interaction is fundamental to human society and shaped by roles, identity, social relationships, inequalities and contexts [1]. The activity leads to meaning creation, which plays a vital role in all encounters performed within healthcare organisations. Encounters between registered nurses, patients and relatives are a common form of social interaction. All involved bring sociodemographic, psychological, cultural and health-related characteristics to the encounter [2]. Social interaction within healthcare is a tool in the patient’s healing process, strengthens social bonds and promotes trust between the parties involved [3]. Therefore, social interaction is central for creating a care relationship, assisting patients and promoting a faster recovery [4] as research demonstrates the way in which nurses interact with patients affects their well-being [5].

Patient satisfaction with social interaction is an important predictor of overall satisfaction with the hospital experience [6]. Older patients sometimes experience difficulties attending and making decisions in the encounter due, for example, to impaired hearing or vision but also to nurses’ lack of time [7]. Therefore, they delegate the tasks of seeking, receiving and providing information to relatives. The presence of relatives can be crucial for supporting older persons in various healthcare situations, as the social interaction includes both an exchange of information and collaboration between patients, nurses and relatives [2]. Relatives can act as social support, as they can reduce the patient’s stress, increase the nurse’s understanding, confirm instructions and agreements, promote communication between family members and work to preserve positive relationships between those involved [8]. However, a third person can prolong the conversation, as more information is usually needed, but also limit the exchange of information as the patient tends to leave the responsibility and social interaction to the relative. Moreover, repeated conversations and social interactions between nurses, patients and relatives result in both patients and relatives feeling more involved in the care [9].

Nurses express those encounters and social interactions with patients and relatives are central for providing care and enriching their work [10]. Knowledgeable and communicative nurses are highlighted as most valuable for patient perceptions of care quality [11], but the healthcare environment and culture are also crucial for a positive care relationship [12]. It has been shown that to ensure the quality of nurse-patient interactions it is important to focus on issues such as power, the social and cultural context and interpersonal competencies [13]. For example, nurses frequently have to ask questions about intimate personal matters. Patients who are highly vulnerable due to a health crisis are obliged to depend upon nurses for their basic needs, which can create an asymmetry and imbalance in the care relationship. Furthermore, healthcare environments are often stressful and limited time can affect interactions and care relationships [14]. As a result, complaints from patients and relatives often concern social interaction [15, 16].

As social interaction is the basis for all social constellations and places high demands on those involved, it can easily lead to misunderstandings [17]. Misunderstandings and problems with social interactions can leave patients confused, dissatisfied and ill-prepared to make decisions or participate in their own care. Misunderstandings can also delay diagnosis of the patient’s problem [16].

In summary, research shows that social interactions between registered nurses, patients and relatives are pervasive, essential and play a central role in healthcare encounters. Lack of interaction and interplay can leave patients confused and ill-prepared to participate in their own care [2], which can threaten patient safety. Thus, social interaction is significant, complex and highlights the importance of understanding. Using Goffman’s social interaction perspective, this study focuses on exploring social interaction in encounters between registered nurses, older patients and relatives at a department of medicine for older people. There are few studies on how social interaction is shaped in such encounters using ethnography as a method and Goffman’s social interactional perspective as a theoretical framework. Research about social interaction is important for deepening the knowledge of how to understand the interaction between registered nurses, older patients and relatives.

## Theoretical framework

Goffman’s interactional perspective uses metaphors borrowed from dramaturgy such as front stage, backstage, performance, audience, roles and framing [18]. Goffman’s texts describe social interaction between individuals and how it is performed to protect a desirable image, using the theatre to illustrate the differing front stage and backstage behaviour. The performance is carried out on the front stage, which is formal and restrained in nature. The actor is conscious of being observed by an audience. The audience is aware of the performance they are participating in, where the actors play given roles with certain rules. Accordingly, each actor tries to convey a picture of her/himself in interaction with others [18]. The preparation for the performance takes place backstage, which is a space without an audience for the construction of characters to be presented on the front stage.

Performance describes a situation in which the participants regularly interact with a specific audience [18]. In this study, the encounter can be seen as a performance. Social rituals, for example, greeting rituals, create a common social situation. Ritualization in social interaction means that the interacting participants use a culturally developed and routine signal system to show that the performance is within the framework considered appropriate [1]. The framing stands for the physical environment where the performance takes place. in this study, the system of values and norms is built to provide care, while the framing is seen as a concept that defines a situation and thereby renders the actions and utterances comprehensible.

Definition of the situation concerns how patients, nurses and relatives try to understand and handle the encounter while gathering information and reading the persons they are interacting with. The basis for everyone is expectations of an identical performance in the encounter, where roles and performances are repeated in the same framework and social institutions, which is internalized by the participants as future knowledge. When this happens in defined locations, social institutions are created with predetermined expectations, norms and rules.

The basic idea in the social interactional perspective [1, 19] is that people understand the activities they participate in by using their previous experience of similar situations. Therefore, they define an encounter in various individual ways and have different expectations about how the encounter will be designed.

Goffman’s work has received attention across the healthcare sciences, for example to illuminate interprofessional practice on hospital wards [20]. In the context of nurses’ socialisation, A Pettersson and S Glasdam [21] expanded Goffman’s notion of front- and backstage. Front stage, patients often functioned as objects for newly employed nurses’ communication training, while backstage patients frequently functioned as objects for all professionals. Thus, Goffman’s interactional perspective could be useful for exploring and creating an understanding of the interaction between registered nurses, patients and relatives. Therefore, the aim was to explore the social interaction in encounters between registered nurses, older patients and their relatives at a department of medicine for older people.

## Methods

### Design

The study design was explorative with an ethnographic approach, followed by a thematic analysis with an abductive approach reflecting Goffman’s interactional perspective. A central tenet in ethnography is that individuals’ experiences are socially organized with focus on human interaction and the construction of the interplay between the individuals involved. Ethnography explores the meaning of activity and interaction [22]. Hence it is particularly useful in the healthcare sciences and for investigating the social interaction between registered nurses, older patients and their relatives in encounters in these environments. Thematic analysis was used in this study because it is a method of identifying, analyzing and presenting patterns that can be employed in different contexts. Due to its theoretical freedom it constitutes a flexible and useful research tool, which can potentially provide a rich and detailed account of data [23]. Abduction uses both inductive and deductive approaches. The inductive approach comprised the production of empirical data, while the deductive approach consisted of a theoretical hypothesis about social interactions. The abductive approach alternates between empirical and theoretical approaches and unites them [24, 25]. The social interactional perspective by Goffman [1, 18, 19] served as the theoretical framework.

### Contextual description of the practice

The research field was two wards in a department of medicine for older people in a medium-sized public hospital in a municipality with about 50,000 inhabitants. The design of the wards was similar and each ward had 24 care beds. The department serves persons aged over 75 years with multiple morbidities who need several different types of care. The staff members treat actual illness, run diagnostic tests and plan for patients’ future needs in cooperation with other healthcare providers such as healthcare centres and municipalities.

The department was selected due to its uniqueness in terms of interprofessional work based on the Comprehensive Geriatric Assessment (CGA) method [26] in the care of this target group. The method includes integrated structured care of older people**,** where the team is an integral part of the unit’s operations and has direct responsibility for patients. The staff comprised physicians, registered nurses, occupational therapists, physiotherapists, counsellors and enrolled nurses. Each registered nurse was responsible for about eight patients per work shift.

Special routines to help patients and relatives have been designed. For instance, healthcare centres can refer patients directly to the department, thus bypassing the emergency care department; the patient or relative can phone the ward for help; and an overall assessment is made of the patient’s life situation. Each patient receives a record with contact information and notes from the last care episode to keep for the next time care is needed at the department or in primary care.

### Participants and recruitment

The participants in the study were registered nurses, patients and their relatives (Table 1).

**Table 1:** Participating patients, relatives and nurses

	n	age, range (mean)	male	female
Patients	21	77–96 (87)	8	13
Relatives	21	30–90 (59)	7	14
Nurses	19	23–62 (42)	0	19

The recruitment of participants started after written permission was obtained from the hospital managers and the wards. A formal ethical review process took place. The first author recruited the participants and provided detailed information about the study to nurses at the ward and at nurse meetings. Informed consent forms were left openly available in staff rooms for a month so that the nurses could take their time deciding to participate, after which their signed informed consent was placed in a folder at the wards. The confidential consent forms were collected by the researcher and stored in a safe place. The participants were all registered nurses who had been working at the wards for between six months and seven years. Of the 42 nurses who provided informed consent, 19 were included in this study because they participated in encounters with patients and relatives. Two of the nurses were observed twice due to illness and changing work shifts.

Patients with visiting relatives were contacted individually and received verbal and written information about the study. Patients identified as critically ill by the ward manager were excluded for ethical reasons. All patients and relatives who were contacted agreed to participate and signed their informed consent, four patients in audio-recorded form because of paralysis. Relatives were children, partners, sons/daughters-in-law, or friends.

### Data collection

Before starting the data collection, the first author spent two weeks with the ward staff to get to know the ward, nurses, routines and to become a familiar face on the wards. The author had no relationship with the unit staff before, during or after the data collection. To harmonize, the author dressed in the same way as the regular staff as recommended [22]. The participants were well informed about the author’s presence.

Data collection took place from October 2015 to September 2016 and involved participatory observations (*n* = 21), which enabled the first author to see the social interaction between the participants, and informal field conversations (*n* = 63), which increased understanding of the context. These methods were considered suitable for exploring social interaction in encounters [22]. The strength of the various techniques is that they provide the opportunity to see and experience the situation. In each of the conversations the three actors (i.e. registered nurses, patients and their relatives) contributed comprehensive information when asked about the social interaction. The social interactions observed were both planned and unplanned and constitute front stage performances. Accordingly, they were mostly structured activities that take place in a public area.

The participatory observations and informal field conversations were audio-recorded and comprised 110 h of data. Field notes with reflections were also written during the observations. In order to obtain as complete a picture as possible, each participatory observation lasted from 30 to 90 min and took place at different times, days and locations such as patient rooms and meeting rooms [22].

After each participatory observation, informal field conversations (approximately 15–25 min) were carried out with the patients, relatives and registered nurses (*n* = 63). The informal field conversations were audio recorded. Open questions were posed about the encounter such as: *Can you tell me about the previous encounter and how you experienced it? What happened...? Can you explain how...?* The data were summarized to give the participants the opportunity to make further comments. The informal field conversations with patients and relatives took place individually in each patient’s room or a special meeting room depending on their wishes, while those with nurses were carried out at locations chosen by the nurse (e.g., staff room, report room).

The conversations were conducted in Swedish, after which the text was translated by the authors and proofread by a professional translator. The focus was on the content rather than on translating verbatim.

### Data analysis

The data analysis was conducted using thematic analysis [23] with an abductive approach [25]. Thematic analysis is a method of identifying and analysing patterns and can be used in different contexts [23].

The analysis process was governed by the aim, the theoretical framework and the perspectives of the registered nurse, older patient and relative. Initially, the transcribed data from participant observations, field notes and informal field conversations were read and re-read with particular attention to the social interaction between the parties involved [23]. Two questions created from the theoretical framework drawn from E Goffman’s [27] interactional perspective were posed to the data; *How do the nurses, patients and relatives define the situation?* and *What characterizes interaction at the beginning, during and ending of the encounter?*

The next step was to look for initial codes by data reduction and documenting where and how patterns occurred [23]. The coding process was performed with an abductive approach [24, 25] to include the central social interaction in the encounter. In the third step, the codes were organized. Similar codes were combined into groups from which a main theme with subthemes was developed. Thematic maps were written manually and used to help with the development of themes as recommended by V Braun and V Clarke [23]. In the fourth step, we ensured that the main theme was uniform and distinct, while in step five it was named and defined. In each subtheme nuances describing different aspects from the perspectives of nurses, patients and relatives were highlighted. Throughout the steps, a back-and-forth movement in the data was necessary as the analysis was a recursive process. Finally, the text was embedded to demonstrate the prevalence of the theme and strengthened by the inclusion of citations and explanations based on the theoretical framework [23].

### Ethical considerations

The ethical review board in Gothenburg approved the study (Ref: 584–15). The participants received verbal and written information before providing their informed consent to participate in accordance with the Declaration of Helsinki [28]. The information stated that participation was voluntary and that they could withdraw from the study at any time. The patients and relatives received information that the patient’s care would not be affected if they did not want to participate or chose to withdraw from the study. Information was also given about the audio-recordings associated with the participatory observations and informal field conversations, the study aim, methodology and how the results would be presented in a manner that would protect their identity. The confidential information collected during the study was stored in such a way that unauthorized persons were unable to access it.

### Trustworthiness and limitation

The combination of various data collection methods made it possible to gain greater insight into the different perspectives of registered nurses, older patients and their relatives. Comparison of data collection techniques also constitutes a basis for checking interpretations [29]. Participatory observation implies the risk of the researcher’s presence affecting the encounter as the observed persons may modify their behaviours in response to the knowledge of being observed, the so-called Hawthorne effect [22, 29], but becoming a familiar face can reduce this risk. Patients and relatives are in a dependency situation and the nurses may make some extra effort due to the knowledge of being observed. To reduce this risk, the author was present at the ward for two weeks before the study started to become a familiar face. The informal field conversations were performed in direct connection with the interaction, which meant that the participants had a clear memory of it, thus strengthening the trustworthiness. Dependability was enhanced by the fact that the observations took place at different times, in various places on the ward and on different days [22]. In terms of credibility, a detailed description of the method, participants, setting, data collection and results has been provided. In the results quotations were used, which strengthens confirmability [30]. The research team was cross-professional and the analysis was critically discussed as teamwork.

Data collection from only two locations is a limitation, thus the findings are more appropriate for achieving a conceptual understanding than for generalization. The transcripts were not returned to the participants for comments or correction, which could be another limitation. However, the participants’ statements were summarized during the informal field conversations, giving them an opportunity to confirm or dispute the content in line with Silverman [29].

## Results

**Fig. 1:**
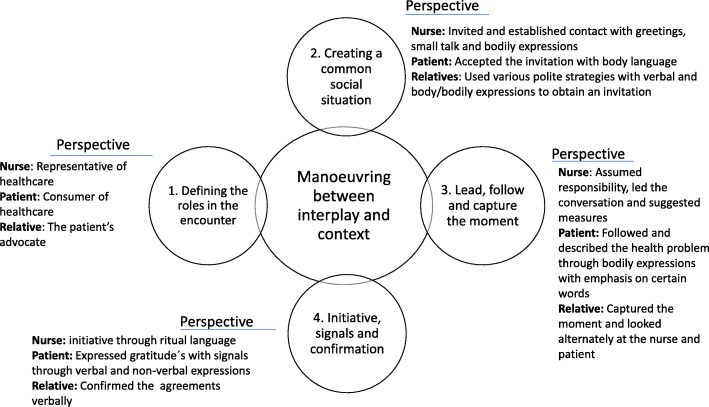
Summary of social interaction in encounters between registered nurses, older patients and relatives

The social interaction between registered nurses, older patients and their relatives revealed a pattern where the participants manoeuvred between interplay and context. By manoeuvring, they defined the roles, created a common social situation, led, followed and captured the moment to receive and provide information. Finally, the interaction contained initiatives, signals and confirmations. The results are presented with subthemes including embedded perspectives of nurses, patients and relatives (Fig. 1).

### Defining the roles in the encounter

Registered nurses, older patients and their relatives expressed an idea of how the social interaction would occur based on their previous knowledge of their respective roles as a nurse, a patient and a relative. At the beginning of the encounter everyone involved was aware of the intention, i.e., to talk about the patient’s health process. Even though they began the encounter from various perspectives, they all defined their roles similarly.

The nurses’ definition was that they represented healthcare and had a professional role. In their work as a healthcare representative, they would provide information to the patient and her/his relatives about the patient’s condition, test results and the planned duration of care. They would also ask for information relevant to the patient’s care. The nurses considered the patient central and the relatives a resource.

Well, I mostly turned to the patient. It is the patient who is in focus, who I give information about tests and plans for the future. The relative is a helping hand of course, but the patient is the main character (Nurse 10).

The patients saw themselves as a person in need of care, a consumer of healthcare. Their role was to provide the nurse with information about how they were feeling and to answer the questions about their social situation. The encounters were sensitive for the patient when it came to her/his health situation and future health, therefore they sometimes asked their relatives to represent them.

I have concerns [points at her heart]. I want them [the nurses] to know about my case. I hope to get answers to my questions and I have three children who ask questions if I tell them to (Patient 19).

The relatives’ definition was that they should act as the patient**’**s advocate when she/he needed them. This role could be initiated by the patient, the nurse or the relative her/himself.

My dad asked me and relied on me to be his stand-in and ask the nurse questions and answer any questions asked. He has hearing difficulties and therefore problems understanding what is said (Relative15).

Sometimes the relative was excluded during the conversation by the nurse. The encounter was consequently not experienced as satisfying by the relative.

The nurse did not see me. Did not talk to me at all. I felt lost (Relative 14).

The nurses, patients and relatives all had experience of social interaction in healthcare, which explains why each defined the situation similarly. Typical of a total institution is that the department is considered to belong to the staff. If the parties share this view, it seems to govern what happens and what does not happen, what is perceived and what is not reflected on [1]. Experiences of previous encounters were brought into the new meetings by the participants. This means that the parties share a common knowledge base concerning how an encounter is performed in hospitals. The experience contributes to and builds on mutual cultural knowledge based on a common understanding of the situation [1]. The cultural knowledge contains different rules of conduct about how participants should behave towards each other and all adopt various roles with different functions.

We all take different roles in social interaction with other people [18]. Role identity can be described as a person assigning her/himself a role. Everything associated with the role is seen as its identity. A description of the role suggests that the individual always assumes a role and that people have several different roles depending on the audience. Different roles can be seen as tools to convey an impression of oneself to the audience and individuals constantly strive to present a genuine and credible image. When continuously played in front of a specific audience, for example an older patient, the role lays the foundation for social connection. The connection means that the role must continue to be played and maintained because the social connection has created expectations of the role [18]. In the present study, the patient’s role was a consumer of healthcare, the relative’s role was the patient’s advocate and the nurse’s role was a representative of the healthcare system. These roles were part of the starting point, along with their previous experiences and expectations. When a person identifies with a role in an encounter, she/he has an obligation to behave according to the moral rules of conduct assigned to the role, which corresponds to the expectations of the others [19]. When the rules of conduct become a routine and ritual act, they become institutionalized and thus part of the institutional order [1]. At the same time, the institutional order is based on the specific context in which the encounter takes place.

By repeating roles and appearances within the same framework and social institutions, the institutional order is internalized in the actor and the audience as knowledge that in the future forms the basis for everyone’s expectations of the same performance within the same social institution. The role becomes social and partially stereotypical. When this takes place, social and other institutions are created with predetermined expectations, norms and rules [1].

As professionals, the registered nurses were used to the healthcare environment, which was their workplace. The older patients were often familiar with the healthcare environment and tried to adapt. For relatives, the healthcare environment was sometimes new or unknown. A social institution consists of a front region and back region [18] and can be compared with the healthcare environment. Where nurses, patients and relatives meet and interact is considered the front region, e.g., the patient’s room. Here, expectations and norms were maintained and a formal relationship existed. In this study, the nurses were the main actors, but they asked the patient or relatives to describe the situation related to the patient’s health process. Relatives were sometimes excluded from the conversation and did not feel welcome, but it also happened that the nurse and the relative excluded the older patient. At such times, misunderstandings could easily occur and affect the encounter in a negative way, which was obvious in the glances exchanged between those involved.

Nurses described how they prepared themselves, for example by reading test results or other documentation. The performance of routines and roles is prepared in the back region [18]. The back region could be the staff room or nurses’ report room where the registered nurses have spatial privacy and informal roles. Spatial privacy for older patients and their relatives could be in the patient’s room with a closed door.

### Creating a common social situation

At the beginning of an encounter, the parties involved tried to create a common social situation. The registered nurses, older patients and relatives manoeuvred towards each other by means of a pattern of actions such as small talk and facial expressions, greetings, smiles, nods, winks, eye contact, a friendly tone of voice and turning to each other.

A repeated pattern was that the registered nurse initiated and began the social interaction by a greeting to invite the participants into the encounter. The registered nurse tried to establish eye contact with the patient and smiled, nodded, or waved when approaching the older patient and relative, regardless of where the encounters occurred. The presentation and greeting were similar: *“Hi, my name is … and I work as a nurse at this ward. How are you today?”* When the patient was sitting on a chair the registered nurse stretched out her hand towards the older patient and greeted. When the patient was lying in bed, the nurse greeted by placing her hand on the patient’s arm or hand and bending forward to maintain eye contact. There was some small talk before the nurse turned to the relative, tried to establish eye contact and greeted by stretching out her hand. The nurse constantly used the patient’s name in the conversation.

The patient sits in the bed. The nurse walks forward, nods to the relative, smiles. Lays her hand on the patient's arm, bends forward, speaks a little louder and tries to make eye contact (Field note 5).

The older patients turned to the registered nurse, tried to establish eye contact, focused on the nurse and accepted the invitation with a nod. If the older patients were sitting on a chair, they got up and greeted by shaking the registered nurse’s hand and then sat down again. Those who could not get up waved and smiled back at the nurse. If the patients were in bed and unable to get up, they asked the relative for help.

The patient looks up when the nurse comes and tries to stand up, but the nurse prevents the patient by showing with her hand that the patient should sit down. The patient looks at the nurse and she smiles (Field note 14).

The relatives used various polite strategies to obtain an invitation, for example, when the nurse entered the room they stood up (if they were sitting) but waited for the registered nurse to change her focus from the older patient before trying to establish eye contact. Then they sat down and waited until they were addressed. Relatives who stated that they had experience of healthcare took a more active role and sometimes invited themselves without waiting for signals from the nurse. Other relatives chose to focus their attention on the patient and spoke with her/him.

I usually do not give up. I try to reach them [the nurses]. I have some experience with my work. Even if I sometimes see them running and in a hurry. I do not give up and this time I asked before she [the registered nurse] could tell (Relative 10).

Each encounter requires an opening that shows inattention is over and focused attention is initiated [19]. Initially, the registered nurses invited and similarly greeted everyone, which was considered polite. It was an everyday routine, which is part of a formal meeting in a healthcare setting. Registered nurses, older patients and relatives all nodded, smiled at each other and sometimes waved. Patterns of verbal and nonverbal action were developed, where the participants expressed their view of the situation [1]. The actor is often unaware of how much of her/his performance is routinized [18]. Greeting rituals create a common social world. Ritualization in social interaction means that the interacting persons use a culturally developed routine signal system to show that the performance is within the framework considered appropriate [1]. Registered nurses used the older patient’s name when talking to her/him. This was a way of removing the mask of anonymity, an acknowledgement of connection [18].

### Lead, follow and capture the moment

During the encounter, the registered nurse, older patient and their relative changed the order of the interaction and roles, where everyone could alternately speak and be in the main role. A repeated pattern was that the participants reinforced or clarified their reasoning with the help of hand gestures, facial expression, or other body movements.

As a healthcare representative, the registered nurse assumed responsibility for leading and keeping the conversation going, suggested measures but also controlled the content of the conversation. Metaphorically, the registered nurse was holding a compass to steer in the right direction. When the registered nurse turned to the older patient the conversation was more about personal care questions, but the same pattern occurred. When the registered nurse turned to relatives, these confirmed that they understood what to do, how the medication should be given or how things work by nodding and waving. The registered nurse often took a position between the older patient and the relative, bent down to be on the same level as the patient, or sat on a chair. While the registered nurse was trying to understand the older patient, she kept the conversation flowing by orienting herself towards the patient’s problem and history. By looking at the patient, the nurse noted her/his general condition, for example, skin colour, sweating, movements, but also reactions. The nurse tried to establish eye contact with the patient and temporarily with the relative.

I look at the patient. How they look, skin colour. Is the person worried? Then I use body language and touch. I point, does it hurt there? Or facial expressions (Nurse 21).

The older patient followed the conversation and told her/his story when the registered nurse gave her/him the opportunity to take on the main role. In their story, the older patients emphasized certain words more strongly, pointed and showed where the problems were located. The patients had previous experiences of healthcare and talked about them in an attempt to objectively describe their situation. The older patients were often passive, waited to be invited and used various aids such as notes when interacting.

Relative: She's in pain.

Nurse: (Turns to the patient) Do you have leg pain?

Patient: Yes, it hurts here (Points with her hand over the hips to the back). It started yesterday. Have had pain there before but it was many years ago, 1985 (She makes the same movement again with both hands) (Observation 1).

Relatives directed their gaze to the registered nurse during the conversation to capture the information given. They also prepared themselves to help the older patient and assumed the main role themselves when necessary. To do so, they followed the conversation closely, their gaze alternated between the nurse and the patient and they nodded, smiled and captured the moment by asking questions when they got the registered nurse’s attention. In this way, they could lead the conversation for a moment and then give the responsibility back to the registered nurse by asking a question.

Sometimes they [nurses] talk so fast. I have to think about what to ask before I come here and then capture the moment when it occurs (Relative 20).

A prerequisite for keeping the interaction going is remaining focused, which means that the parties pay extra attention to each other and confirm each other, thereby giving everyone an appropriate opportunity to work towards a common goal [18]. Everyone directed their attention to those who were currently speaking. Utterances like yes, hmm, or no are used. Social relationships are vulnerable and dependent on what happens in the encounter. If the rules are broken, a feeling of unease will be experienced, leading to negative sanctions on the behaviours. Misunderstandings easily arise about the nature of the encounter due to lack of communication or false expectations. By paying attention to what the patient and relatives said, the nurse quickly changes the orientation when they begin to digress.

### Initiative, signals and confirmation

The registered nurses initiated the end of the encounter by ritual language and actions. The older patient and relative understood what was going on and gave signals by using similar expressions. They confirmed, nodded, smiled and used words like yes, well, I know or precisely.

The registered nurses ended the encounter by summarizing or repeating what had been said. They indicated by their actions that the encounter was over by, for example, getting up (if sitting), folding the bedside table or taking some steps back. Finally, the registered nurses also encouraged the older patient and gave hope by means of small talk. It sometimes happened that on the way out the nurses asked if the patient had any further questions.

Nurse: Well, this sounds very fine, I will come back later, but again, great that you feel better. On Monday there is time for new tests. Just ring the bell if there is something you want to know [nurse smiles, nods and waves (Observation 12).

The older patients mostly expressed their gratitude with both verbal and nonverbal expressions. They thanked the nurse for the attention they had received and some patients made a joke, following the nurse with their eyes for confirmation. They also complimented the nurse, despite sometimes experiencing that the nurses were in a hurry and did not have time for them.

Yes, they have a lot to do but she [the nurse] is a lovely person. She was so happy, nice and polite (Patient 3).

The relatives focused on the conversation. They tried to encourage without intervening, followed the one who was talking with their eyes and nodded. When the registered nurse indicated that the conversation was ending, they got up if they were sitting. The relatives often asked one last question to clarify the agreements.

Daughter: [Standing up] And does she get antibiotics tonight too? How long does she get that? What did you say about that?

Nurse: Yes, she gets it at four and ten o'clock. Until the end of this week (Observation 2).

Ending an encounter requires an initiative and signals [19] and the registered nurse was the one who took that initiative. The older patient and relatives focused on the nurse through eye contact and active listening. They used facial expressions to show their focused attention in addition to nodding. They also engaged in small talk with each other. Those involved must assess the overall impression to define the situation and thereby make appropriate response [18]. Individuals read each other continuously to gain knowledge of how the others are to be perceived, while their previous experiences from earlier encounters are brought into play and contribute to a common understanding of the situation [1].

## Discussion

The study contributes knowledge and understanding of social interaction between registered nurses, older patients and relatives at a department of medicine for older people, through the lens of Goffman’s interactional perspective. The study adds a three-part perspective of social interaction in the encounter. The result is important for understanding the complexity of social interaction and explains why misunderstanding could occur when three people meet.

The results show that the participants had a picture of how an encounter takes place. In this context, they seemed to have a common cultural base within the interactional order that contains rules of conduct, i.e., how to behave politely towards each other. For example, rising from the chair and greeting when someone enters the room, as both the older patients and relatives did, is a courtesy ritual learned from previous social meetings. It is commonly understood that social interaction and behavioural norms are culturally specific and also related to gender and age [1], all of which are a part of the context at a department of medicine for older people.

The registered nurses, older patients and relatives manoeuvred in line with a given structure in the front stage, the encounter, which had a beginning and an end [19]. One can also discern the conversation phases with an introduction, a central part and an end [17].

The registered nurses usually initiate the interaction and are involved in the largest proportion of staff-patient interactions at hospital wards [31]. This study showed that the nurse was the healthcare representative, the person who initiated, invited and established the encounter but also assumed and carried the responsibility to lead it and create a balanced care relationship. Most of the responsibilities were considered a work task, which was done both consciously and unconsciously. The nurses’ leader position related to their profession causes asymmetry, leading to imbalance in the encounter between those involved. An imbalanced relationship can be both increased and toned down depending on [19], in this case, the registered nurse’s approach and way of conversing with older patients and relatives. Attention, kindness, listening and awareness seem to be important aspects that increase the possibility of creating a satisfactory care relationship together [32]. A registered nurse’s approach is crucial and constant training in communication and social interaction skills is important.

The results indicated that smiling, nodding and waving seemed to be an effective universal language that all understood. Many older persons have problems with hearing, vision and understanding concepts, which lead to difficulty comprehending and responding to nurses’ questions or even having a conversation [33]. In this study, the older patients were often passive, waiting to be invited to the encounter, which could be age or health related, but also due to experiences of previous social interactions [1]. Many older patients are also afraid of being a burden to, for example, nurses or relatives [34]. A prerequisite for maintaining social interaction is that the parties involved pay attention to each other’s performances and confirm each other [1]. In this study, the patients expressed gratitude for the attention they received, even if it was just for a couple of minutes. The encounter could sometimes be delicate and they asked their relatives for support, which is something they are expected to do as part of the definition of the situation [1].

The results showed that relatives used smiling, nodding and rising from the chair to obtain an invitation to the conversation and interaction, they clarified the content to eliminate ambiguities, for example, post-stroke. Relatives want to be confirmed as a family and have a trusting relationship with and be informed by the registered nurses so that they can provide support at home [4].

The study revealed that the parties involved tried to create a common social situation with both verbal and non-verbal communication and interaction. Clear and honest social interaction leads to fewer misunderstandings. Satisfied older patients and relatives are more likely to follow advice and instructions [35], which can help to develop a satisfactory care relationship between the three parties.

## Conclusion

The results provide knowledge about social interaction in healthcare encounters from three perspectives: nurses, patients and relatives. The social interaction was shaped by a pattern where the participants manoeuvred between interplay and context. By manoeuvring, they defined roles, created a common social situation, led, followed and captured the moment. When all participants assume responsibility for the social interaction in the encounter, it makes them listen more actively, thus reducing the imbalance and misunderstanding so that a satisfactory care relationship can be achieved, which is important for ensuring safe care.

An awareness of the complexity and importance of social interaction in an encounter gives the registered nurse an understanding but also a tool to approach the patient and relatives in order eliminate any misunderstandings that have arisen and thus provide safe care. Nurses’ approach is crucial and constant training in communication and social interaction are important.

### Implication for nursing practice

The findings show the importance of an awareness of the meaning of social interaction in encounters between registered nurses, patients and their relatives. Registered nurses are in a unique position to initiate and lead the conversation, using a holistic approach to improve patients’, relatives’ and even their own understanding of the information provided by all persons involved. Therefore, the findings can be used as a tool in training interventions for improving social interaction in healthcare organisations, such as reflecting teams at hospital wards. The knowledge can also be used in the context of nursing education and nursing students’ clinical learning, for example in communication with patients and relatives.

## Data Availability

Additional datafiles in Swedish are available upon request to the corresponding author.
